# Evaluation of nationwide analysis surveillance for methicillin-resistant Staphylococcus aureus within Genomic Medicine Sweden

**DOI:** 10.1099/mgen.0.001331

**Published:** 2025-01-27

**Authors:** Erika Tång Hallbäck, Jonas T. Björkman, Fredrik Dyrkell, Jenny Welander, Hong Fang, Isak Sylvin, René Kaden, Hinnerk Eilers, Anna Söderlund Strand, Sara Mernelius, Linda Berglind, Amaya Campillay Lagos, Lars Engstrand, Per Sikora, Paula Mölling

**Affiliations:** 1 Department of Infectious Diseases, Institute of Biomedicine, Sahlgrenska Academy, University of Gothenburg, Gothenburg, Sweden; 2 Region Västra Götaland, Sahlgrenska University Hospital, Department of Clinical Microbiology, Gothenburg, Sweden; 3 Center for Molecular Diagnostics, Department of Clinical Genetics, Pathology and Molecular Diagnostics, Office for Medical Services, Region Skåne, Lund, Sweden; 4 1928 Diagnostics, Gothenburg, Sweden; 5 Department of Clinical Microbiology, and Department of Biomedical and Clinical Sciences, Linköping University, Linköping, Sweden; 6 Department of Clinical Microbiology, Medical Diagnostics Karolinska, Karolinska University Hospital, Stockholm, Sweden; 7 Bioinformatics Data Center, Core Facilities, Sahlgrenska Academy, University of Gothenburg, Gothenburg, Sweden; 8 Department of Medical Sciences, Clinical Microbiology, Uppsala University, Uppsala, Sweden; 9 Department of Laboratory Medicine, Clinical Microbiology, Umeå University Hospital, Umeå, Sweden; 10 Clinical Microbiology, Infection Prevention and Control, Office for Medical Services, Region Skåne, Lund, Sweden; 11 Laboratory Medicine, Jönköping Region County, Jönköping and Department of Clinical and Experimental Medicine, Linköping University, Linköping, Sweden; 12 Laboratory Medicine, Jönköping Region County, Jönköping, Sweden; 13 Department of Laboratory Medicine, Clinical Microbiology, Faculty of Medicine and Health, Örebro University, Örebro, Sweden; 14 Department of Microbiology, Tumor and Cell Biology, Centre for Translational Microbiome Research, Karolinska Institute, Solna, Sweden

**Keywords:** cgMLST, Genomic Medicine Sweden, MRSA, SNP, whole-genome sequencing

## Abstract

**Background.** National epidemiological investigations of microbial infections greatly benefit from the increased information gained by whole-genome sequencing (WGS) in combination with standardized approaches for data sharing and analysis.

**Aim.** To evaluate the quality and accuracy of WGS data generated by different laboratories but analysed by joint pipelines to reach a national surveillance approach.

**Methods.** A national methicillin-resistant *Staphylococcus aureus* (MRSA) collection of 20 strains was distributed to nine participating laboratories that performed in-house procedures for WGS. Raw data were shared and analysed by three pipelines: 1928 Diagnostics, JASEN (GMS pipeline) and CLC-Genomics Workbench. The outcomes were compared according to quality, correct strain identification and genetic distances.

**Results.** One isolate contained intraspecies contamination and was excluded from further analysis. The mean sequencing depth varied between sites and technologies. However, all analysis methods identified 12 strains that belonged to one of five outbreak clusters. The cut-off definition was set to <10 allele differences for core genome multilocus sequence typing (cgMLST) and <20 genetic differences for SNP analysis in a pairwise comparison.

**Conclusions.** MRSA isolates, which are whole genome sequenced by different laboratories and analysed using the same bioinformatic pipelines, yielded comparable results for outbreak clustering for both cgMLST and SNP, using the 1928 analysis pipeline. In this study, JASEN was best suited to analyse Illumina data and CLC to analyse within respective technology. In the future, real-time sharing of data and harmonized analysis within the Genomic Medicine Sweden consortium will further facilitate investigations of outbreaks and transmission routes.

**Impact Statement**

Implementing whole-genome sequencing (WGS) in healthcare will enable fast and reliable typing, which in turn increases the possibility of detecting the spread of infectious diseases and antimicrobial resistance. The rapid development of sequencing technology, including bioinformatics and data interpretation, facilitates pathogen sequencing and its potential for clinical use. However, the impact of the different analysis pipelines, even for the same species, is not widely tested.

The results of this study contribute to the community by (i) showing that a harmonized data analysis approach gives comparable results and outbreak cluster definitions even though the sequencing data are produced differently, (ii) paving the way for implementing WGS into routine diagnostics to get a national equal precision diagnostic and (iii) highlighting that the advantage of building national consortiums with joint IT infrastructure and analysis pipelines that analyse sequences generated from different sequencing platforms in real time would facilitate outbreak investigations and transmission routes and also prepare and facilitate for further international data sharing. We here bring light on the necessity of using harmonized and standardized bioinformatic solutions to get reliable and comparable results to improve future outbreak surveillance.

## Data Summary

All supporting data, code and protocols have been provided within the article or through supplementary data files.

Qiagens analysis suite CLC genomics workbench - https://digitalinsights.qiagen.com/

The web based bacterial analysis software created by 1928 diagnostics - https://app.1928diagnostics.com/

The free and open source bacterial analysis pipeline created within the Genomic Medicine Sweden initiative - https://github.com/genomic-medicine-sweden/jasen

## Introduction

The rapid spread of antimicrobial-resistant bacteria is a global concern and significantly contributes to healthcare-associated infections with an increased risk of mortality [1,3]. Implementing next-generation sequencing (NGS) technology in healthcare will enable a fast and reliable identification of these microorganisms, which in turn would increase the possibility of detecting the spread of infectious diseases and antimicrobial resistance. The rapid development of NGS, including bioinformatics and data interpretation, facilitates pathogen sequencing and its potential for clinical use [4,7]. Today, several hospital laboratories are using whole-genome sequencing (WGS) with NGS technology for epidemiological outbreak detection, typing and surveillance of bacteria such as methicillin-resistant *Staphylococcus aureus* (MRSA) and *Mycobacterium tuberculosis* [7,15]. The usage of NGS requires new technical solutions and an informatics infrastructure for data storage and data processing at hospitals. The Genomic Medicine Sweden (GMS) consortium has been established with the aim of implementing high-throughput sequencing technologies and genomics in healthcare to improve precision diagnostics [16]. Within GMS, an infrastructure called the National Genomic Platform (NGP) is being established for handling large-scale sequencing data and computation. The NGP facilitates storage and analysis and aims to share real-time data between the hospitals and the Public Health Agency of Sweden (PHAS). Although the current publication focuses on the evaluation of data analyses for this pilot, with a view to implementing a national surveillance approach for MRSA at the NGP in Sweden, the use case can be expanded to other agents of interest within clinical bacteriology and virology. Outbreak investigations are enhanced by enabling rapid sharing of WGS data generated from different laboratories and sequencing technologies. However, data need to be analysed using the same standardized pipeline to obtain comparable results, as demonstrated by Coolen *et al*., who found discrepancies in reported outbreak clusters between centres using different data analysis approaches [17]. Also, several methods are to be considered for depicting the relatedness of bacterial strains using WGS data, such as core genome multilocus sequence typing (cgMLST) or SNP analysis. Both cgMLST and SNP have their benefits. cgMLST is stable and often sufficient to define an outbreak, but a following SNP analysis could be more discriminative. To facilitate a first step towards a standardized approach, this project was run through the nationwide Genomic Medicine Centres (GMC) in Sweden, including the seven university hospitals, an adjunctive hospital region and the PHAS, with the purpose of determining molecular markers of MRSA for use within outbreak investigations and contact tracing. The clustering of isolates with high genomic similarities within a limited time frame is here defined as a potential outbreak. The investigation was done through a panel of 19 valid MRSA strains, sequenced at all nine sites and analysed by comparing three joint bioinformatics approaches on all sequencing data.

The aim was to evaluate the quality and accuracy of WGS data generated by different sequencing procedures but analysed in joint analysis pipelines, with a future purpose to reach a national real-time monitoring approach within the GMS consortium for MRSA.

## Methods

### Sample material

Twenty MRSA isolates from the national collection were defined by the PHAS. The isolates were collected during 2016–2018 due to outbreak investigations and were from different individuals (patients with clinical infection or carriers). The PHAS defined six clusters by WGS, involving 14 isolates in total. However, only 19 isolates were valid since intraspecies contamination was further detected in one of the strains (isolate 1) (Table S1, available in the online version of this article).

The isolates were distributed to seven university hospitals with a GMC, as well as to one county hospital. Each laboratory cultured the isolates according to local routines, as further detailed in Table S2 and Appendix S1. No genetic typing or clinical epidemiological data were provided for any of these isolates by PHAS.

### DNA extraction, library preparation and sequencing

Each laboratory extracted genomic DNA from the cultured isolates according to local routines, followed by library preparation and sequencing on a local platform (Tables S2 and S3 and Appendix S1). In total, nine sites (A–I) were responsible for sequencing the MRSA isolates, including two sequencing systems from site E (E1 and E2).

### Sequence data analysis

The sequence data were quality checked (QC) and analysed within the participating sites A–I according to local analysis pipelines (Tables 1 and S3). Additionally, the sequence data from sites A–I were shared and analysed by three different software: JASEN (https://github.com/genomic-medicine-sweden/jasen), an assembly-based analysis pipeline developed within GMS using cgMLST, CLC (Qiagen) with a workflow-based variant-calling SNP analysis and 1928 Diagnostics that uses k-mer analysis pipelines for cgMLST and variant-calling SNP analysis approaches. JASEN, CLC (Qiagen) and 1928 Diagnostics use the nomenclature for sequence typing of clonal complexes, continuously updated from PubMLST.org. Due to the lack of clinical epidemiological data, apart from collection year and previous findings from PHAS, the cut-off to define an outbreak cluster was set to <10 alleles differences for cgMLST and <20 genetic differences for SNP analysis in a pairwise comparison in this study [10,18,20].

**Table 1.** Quality parameters for sequencing of the included sequenced isolates

Site	Analysis pipeline	Sequencing depth (average [min–max])	Average read length	Average insert size	Core gene % (average [min–max])	Missing loci % (average [min–max])	IQR	Species identity % (average [min–max])
**A**	1928	108 [73–228]	279		99.0 [96.7–99.8]			99.5 [99.2–99.8]
	JASEN	147 [91–355]		–	99.92 [99.23–100]	31.92 [24.27–51.92]	0.32 [0.19–0.95]	99.4 [99.08–99.68]
**B**	1928	73 [39–109]	189		98.9 [97.1–99.5]			99.4 [99.1–99.8]
	JASEN	92 [43–152]		199 [163–230]	99.93 [99.74–99.99]	1.21 [0–3.31]	0.42 [0.38–0.53]	99.45 [99.12–99.73]
**C**	1928	165 [143–191]	211		99.0 [97.1–99.8]			99.3 [98.7–99.7]
	JASEN	181 [147–218]		214 [201–228]	99.96 [99.81–99.99]	1.19 [0.06–3.31]	0.38 [0.25–0.44]	99.18 [98.76–99.5]
**D**	1928	120 [33–153]	146		98.8 [93.3–99.8]			99.2 [97.3–99.7]
	JASEN	146 [36–202]		244 [214–255]	99.9 [99.32–99.98]	1.3 [0.06–3.31]	0.44 [0.4–0.56]	99.06 [98.06–99.61]
**E1**	1928	90 [42–156]	271		99.1 [97.1–99.8]			99.4 [98.9–99.7]
	JASEN	102 [43–190]		330 [285–357]	99.96 [99.84–100]	1.18 [0.06–3.31]	0.27 [0.22–0.33]	99.34 [99.06–99.62]
**E2**	1928	194 [121–280]	149		99.1 [97.1–99.8]			99.5 [99.1–99.8]
	JASEN	337 [160–645]		325 [290–345]	99.94 [99.75–99.98]	1.21 [0.06–3.31]	0.2 [0.18–0.24]	99.44 [99.13–99.67]
**F**	1928	215 [72–286]	137		98.9 [96.4–99.8]			99.5 [98.2–99.8]
	JASEN	454 [82–841]		207 [142–341]	99.93 [99.74–99.98]	1.31 [0.06–4.59]	0.34 [0.29–0.45]	99.43 [98.91–99.64]
**G**	1928	105 [67–177]	261		99.1 [97.1–99.8]			99.4 [99.1–99.7]
	JASEN	116 [72–208]		319 [301–351]	99.96 [99.79–99.99]	1.22 [0.06–3.31]	0.26 [0.21–0.29]	99.25 [98.87–99.53]
**H**	1928	56 [47–68]	147		91.0 [80.6–96.4]			99.5 [98.9–99.9]
	JASEN	74 [60–94]		314 [270–377]	98.13 [94.81–99.11]	5.36 [2.85–11.21]	0.82 [0.6–1.06]	99.51 [99.05–99.79]
**I**	1928	41 [24–80]	260		98.8 [96.4–99.5]			99.5 [98.9–99.7]
	JASEN	46 [21–99]		–	99.74 [97.8–99.98]	56.86 [33.86–85.19]	0.35 [0.26–0.48]	99.38 [98.96–99.72]

#### Data analysis – QC and cgMLST with JASEN

All samples were analysed with JASEN, a general microbial analysis pipeline for epidemiological typing, anti-microbial resistance and virulence typing. Illumina reads were not trimmed, while Ion Torrent reads were trimmed with postaln_qc.pl from Sneakernet [21]. *De novo* assembly was performed using SKESA 2.4 (default settings) for Illumina reads, and for Ion Torrent reads SPAdes 3.15.5 (with the options Ion Torrent, careful and single cell) was used. The cgMLST analysis was done by ChewBBACA 3.1 [22] using the core-gene scheme demonstrated by Leopold *et al*. [23]. ChewBBACA is a versatile cg/wgMLST-calling software that uses FASTA files as input, which makes it fast but dependent on a good *de novo* assembly. Due to differences in how the cgMLST scheme was defined and how ChewBBACA’s allele-calling works, JASEN was missing a minimum of 139 loci. The results were adjusted to account for this, considering a total of 1722 core genes.

Missing locus calls were not counted as informative, and minimum spanning trees were generated using Edmonds’ algorithm in GrapeTree [24].

Quality analysis of core genome coverage was performed by mapping the raw sequence data against NC_002951.2 (COL). A coverage of >10× over a specific position is considered enough to call the position securely. Core genome coverage was determined by mapping reads to the reference NC_002951.2 (COL) and masking positions that were not included in the cgMLST scheme. Additional QC data were generated by QUAST. The evenness of coverage was calculated as the interquartile range (IQR), defined as the difference between the number of positions in the third and first quartile in the depth of the mapped sequences against the core part of NC_002951.2 (COL) and further divided by the median [(Q3−Q1)/median]. The fragment insert size was calculated with Picard’s ‘CollectInsertSizeMetrics’. Species identity was analysed with Kraken 2, using the standard database.

#### Data analysis – SNP with CLC, Qiagen

Epidemiological strain typing with CLC including all sites (A–I) was performed in CLC Genomics Workbench 21 (Qiagen). The reference *Staphylococcus aureus* ASM1346v1 (GCF_000013465.1) was used for mapping the sequence reads, followed by local realignment and variant calling (Supplementary S1-A). Reads shorter than 20 base pairs were filtered out, and a neighbour-joining (NJ) algorithm was used for the SNP analysis, requiring variants called at a frequency of 90% and a minimum coverage of 10.

#### Data analysis – cgMLST/SNP with 1928 diagnostics

All the samples were uploaded and analysed on the 1928 platform (1928 Diagnostics, Gothenburg, Sweden). Every participating site had a separate account, and a sharing group was configured to enable joint analysis of the data. Before the downstream analysis, the samples were checked for quality. This included trimming reads on base quality and removing primers. An average coverage of >30 was required for the analysis, except for one participant, where this threshold was lowered to >20 due to their high-volume, low-deviation sequencing workflow targeting a lower average. To rule out possible contaminations or mislabelling, species identification was used, and multiple alleles in the core genome were recorded to enable the detection of intra-species contamination. The 1928 cgMLST method uses a custom-developed allele-calling algorithm based on an alignment-free k-mer approach. The cgMLST scheme for *Staphylococcus aureus* comprises 1704 core genes originating from the NCBI Reference sequence NC_009641.1 (Newman), with a 95% similarity threshold. An SNP-based analysis was also performed to evaluate the clonality of the samples, as described in Werner *et al*. [25].

## Results

In one of the batches of the panel of 20 MRSA strains sent by PHAS, one of the strains (strain 1, Table S1) was found to be contaminated with an additional MRSA strain. Further analysis on this isolate was therefore discarded at all sites. The remaining 19 isolates were analysed according to sequencing depth, fragment read length, percentage of the core genome that was covered and percentage of species identity to *S. aureus*. Furthermore, one isolate sequenced from site I, isolate 10, did not pass the requirement for sequencing coverage on the 1928 platform and was excluded from further analysis in this pipeline (Table 1). Both cgMLST and SNP analyses were used to define the outbreak isolates. Even if the cgMLST method is robust, with a great discriminatory power, it can benefit from a more specific and detailed SNP analysis for an even higher discriminatory power. Independently of sequencing technology, five clusters were defined as I–V by all three analysis methods. Cluster I, the largest, included the isolates 7, 10, 16 and 20. The four remaining clusters all contained two isolates each: cluster II comprised isolates 5 and 14, cluster III of isolates 6 and 19, cluster IV of isolates 13 and 15 and cluster V of isolates 4 and 8. The additional strains were completely unrelated (Table S1).

### Analysis of sequence quality

**Fig. 1.**
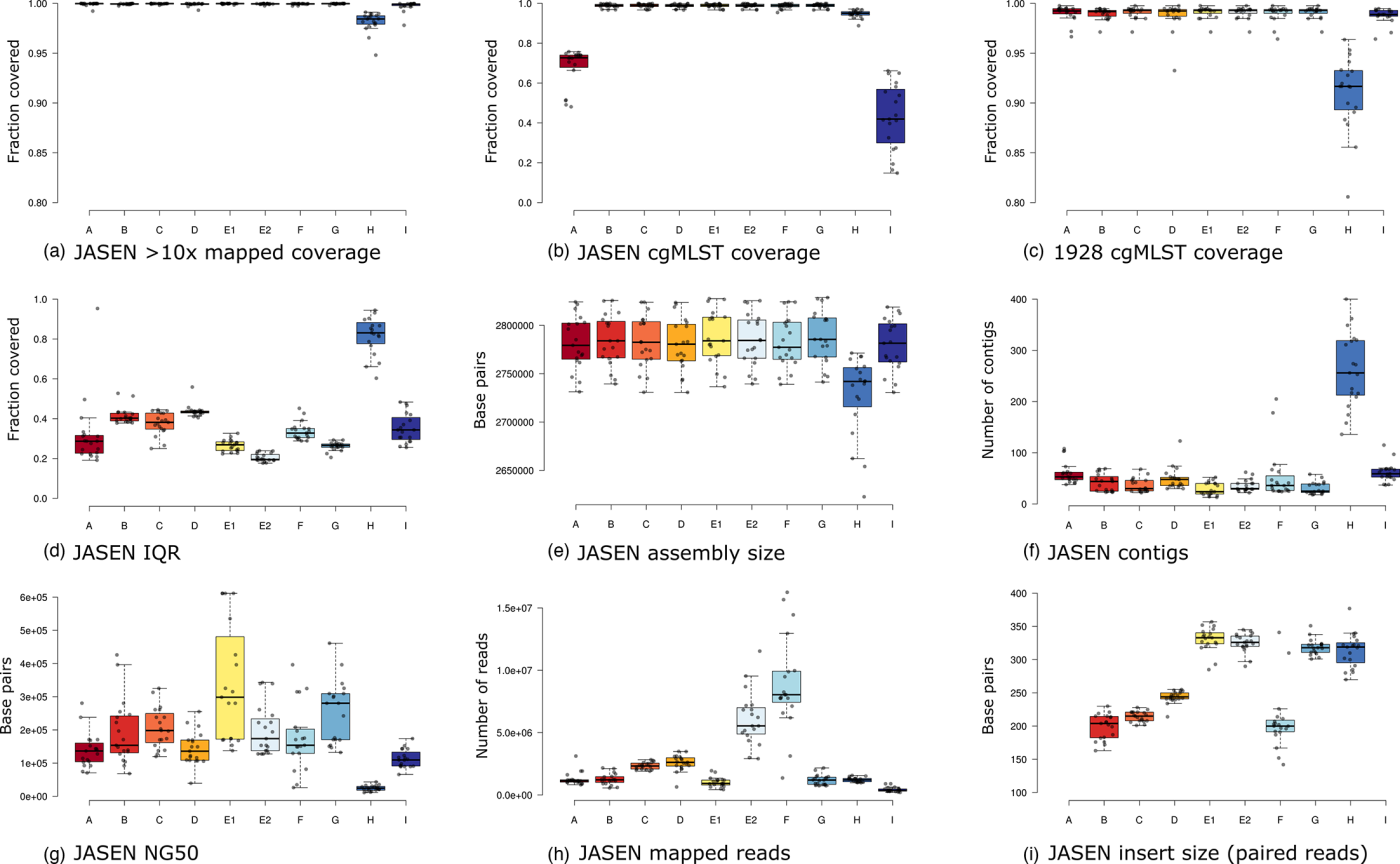
The different figures (**a–i**) show (**a**) coverage by BWA-MEM mapped against the core part of NC_002951.2 (COL), (**b**) percentage of the total number of cgMLST loci detected by chewBBACA in JASEN, (**c**) core coverage for SNP analysis in 1928, (**d**) evenness of coverage presented by showing the IQR of the depth coverage when mapping back reads to the *de novo* assembly, (**e**) *de novo* assembly size, (**f**) number of contigs in *de novo* assembly, (**g**) NG50 values, (**h**) number of total mapped reads to NC_002951.2 (COL) and (i) insert size for paired-end sequencing. All the quality metrics can be found in Table S2.

The quality of the generated sequences was analysed by JASEN and 1928 (Tables 1 and S4, Fig. 1). All sites produced genome sequences with a comparable core coverage (sequencing depth of the core genome), except for site H, which showed a lower quality as well as for site I that for two samples were below the cut-off for sequencing depth to be further analysed for the core genome on the 1928 platform (Tables 1 and S4). Also, the IQR values for the sequences from site H diverged compared to the other sites, meaning that the coverage was more uneven for this participant (Table 1, Fig. 1a). Additionally, comparing sequence quality between the Illumina technologies, the evenness of coverage was sslightly lower for all sites that used Nextera XT (site B, D and H) compared to other library preparation for Illumina sequencing (Table S3, Fig. 1d). No differences in sequencing quality could be correlated to the extraction method used, which is not surprising since all laboratories used well-known extraction methods (Appendix S1).

**Fig. 2.**
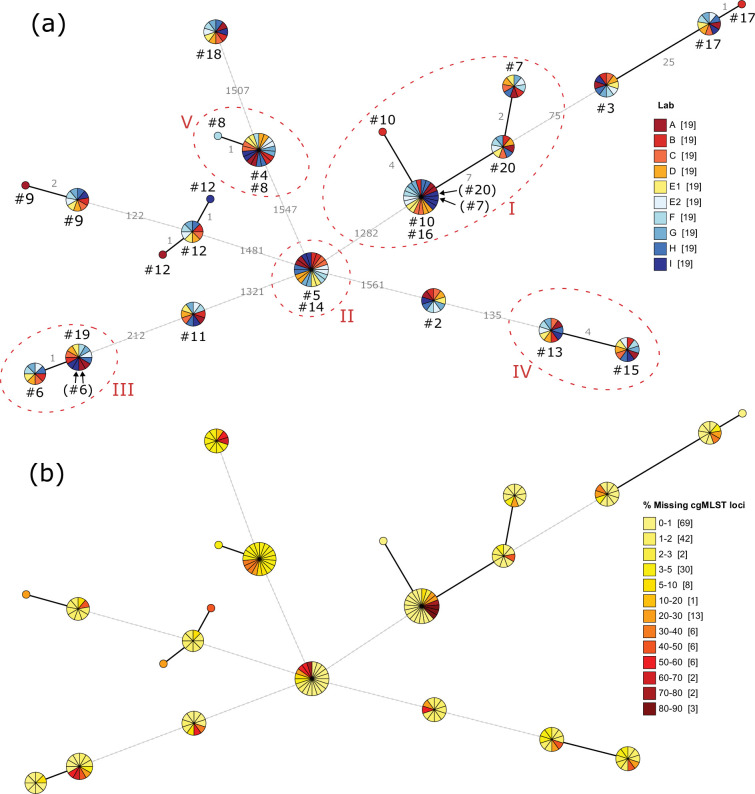
The cgMLST analysis in JASEN was visualized in GrapeTree of the 19 valid isolates (contaminated isolate #1 removed). The dendrogram was created with a minimum spanning tree MSTreeV2 according to Zhou *et al*. [24]. The branches were drawn with a logarithmic length indicating the shorter branches with solid black lines and the longer branches with dotted black lines. (**a**) The numbers of differing loci are indicated above the branches in light grey. The sites A–F are indicated in colours according to the figure and the corresponding sequenced isolate (#). The clusters, CI–CV, are in red circles. Aberrantly clustering isolates are noted with parenthesis and arrows. (**b**) The percentage of successive lack of cgMLST loci is visualized by the colours light yellow to dark red, including the number of sequenced isolates in brackets. See Table S4 for all the data points.

In the cgMLST method of JASEN, a *de novo* assembly was the basis for further downstream analyses. To generate high-quality cgMLST results, the raw reads were filtered stringently, resulting in a lower cgMLST coverage for site A and site I using Ion Torrent sequencing (Fig. 1b). This was especially true for the sequences of lab I, where the amount of data was low. A minimum spanning tree algorithm was used, where samples with missing data groups with the closest matching cgMLST types. For isolates with lower coverage, which are missing the discriminatory loci in a closely related cluster, the isolate can be erroneously assigned to the most common sequence type in the cluster. This was reflected in clusters I and III, where the strains with the lowest coverage were misplaced (Tables 1 and S4 and Fig. 2).

Analyses of the sequences by the 1928 platform generated a cgMLST of high quality for all sites except site H (Fig. 1c). Site H was also the one site to diverge in quality regarding assembly size, number of contigs and NG50 value when analysed by JASEN (Fig. 1e–g).

The number of mapped reads, as shown in Fig. 1h, demonstrated that site F to some extent was able to compensate the low NG50 value due to the high sequencing depth (Table 1). The insert size for sequences generated by Illumina technology also varied significantly from 200 bp to 350 bp. Here, a high sequencing depth could provide some compensation (Fig. 1i, Table 1).

### Analysis results of JASEN

The complete results of the cgMLST by JASEN are presented in Fig. 2. In the largest cluster I, two sequenced isolates from site I, 07 and 20 (marked with arrows in Fig. 2a), lacked 73% and 84% of the cgMLST loci, respectively, and as a result, these sequences clustered with the sequences from isolates 10 and 16. Due to the analysis approach, the resolution in individual clusters will not improve if analysed separately compared to all samples at once.

There were 10 samples in total, which diverged by one or more loci from the other sequences of their participating cluster, with an average distance of 2.3±2.1 loci. Out of these, seven were generated by the Ion Torrent technology. All sequences from these samples were analysed in detail by mapping the raw sequence reads data back to the FASTA file of the divergent locus (data not presented). As a result, four of the sequences generated by Ion Torrent, clustered with another sample; six from site A and six from site I in cluster III and 7 and 20, both from site I in cluster I. Additional sequences with discrepancies produced by Ion Torrent technology were 9 and 12 from site A, which differed in one locus due to a misassembly of 5 adenines to 4 at position 1 349 306. This induced a frameshift and an alternative start codon. Also, 9 from site A and 12 from site I, differed by one allele with a mis-assembly of 3 adenines in position 1 844 079 and an alternative start codon, were detected.

From the Illumina technology, two sequences 10 in cluster I, and 17 (unrelated) from site B, differed by 4 and 1 locus from each sample, respectively, and were both confirmed as true with no ambiguous positions. The sequence 8 from site F in cluster IV, differed by one locus due to low coverage in the start of the core gene, led to the detection of an alternative methionine start codon and hence another allele.

For JASEN, all samples had a maximum distance of 7 loci within a cluster.

### Analysis results of 1928 diagnostics

**Fig. 3.**
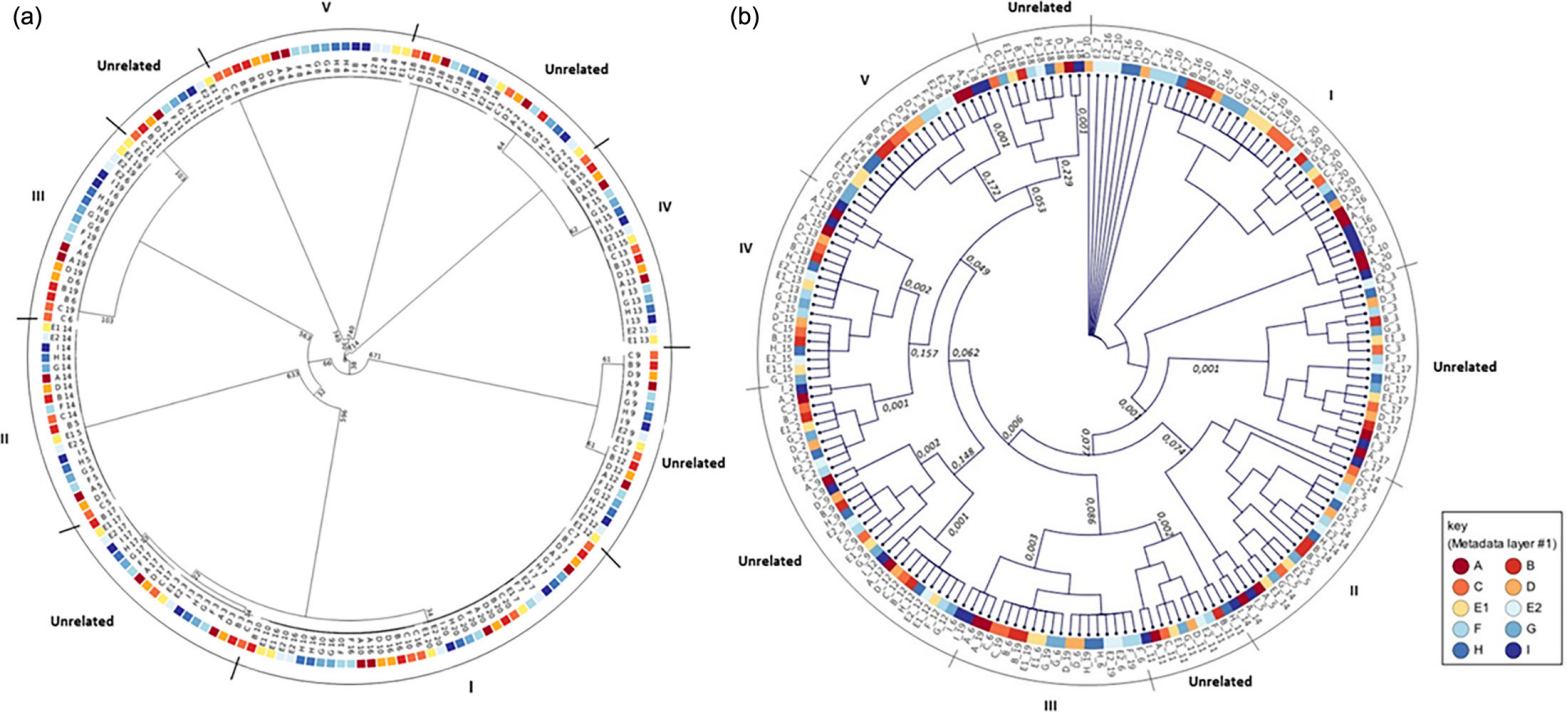
The 19 isolates were analysed based on epidemiological typing and coloured by participants. Contaminated sample 1 removed, E1 – MiSeq, E2 – NextSeq. (**a**) A circular UPGMA dendrogram showing the cgMLST results from the 1928 analysis pipeline. Branch lengths <5 not shown. (**b**) A NJ SNP analysis from the sites A–I. The circular cladogram was performed in CLC by mapping single reads to the reference *Staphylococcus aureus* ASM1346v1 (GCF_000013465.1).

The complete cgMLST analysis of all participants data is shown in Fig. 3a. All sequences for every individual sample clustered together within an allele distance of 0–1, except for sequence 10 from site B. This sequence clustered four allele differences from the other sequences that were identical to no allele differences. As mentioned above, this difference was also confirmed by the JASEN analysis.

**Fig. 4.**
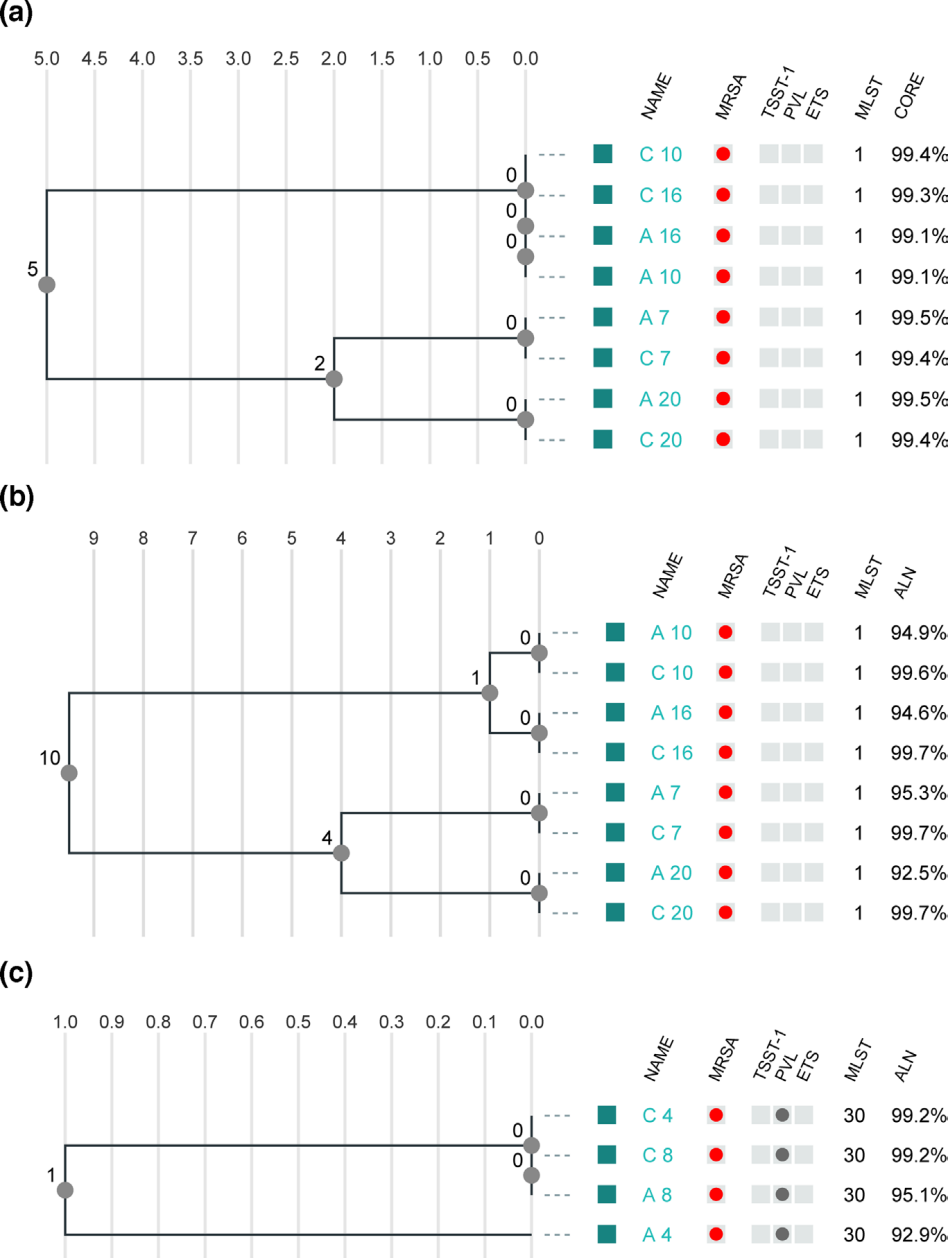
cgMLST and SNP investigation using 1928 analysis pipeline for cluster groups I and V comparing between Illumina and Ion Torrent from participant A and C. (**a**) cgMLST analysis of cluster I, (**b**) SNP analysis of cluster I using the reference genome RefSeq GCF_003724015.1 and (c) SNP analysis of cluster V; RefSeq GCF_900251255.1. No filtering for putative recombination was performed.

The two sites A and C were selected for further comparison of selected clusters (Fig. 4) due to the different sequence technologies from Illumina and ThermoFisher. Furthermore, site A represented the site producing the highest quality of Ion Torrent sequences compared to site I. This was also the case for site C in comparison to additional sites using Illumina sequencing technology. The outbreak cluster I showed the largest diversity, consisting of samples 7, 10, 16 and 20. The cgMLST analysis produced an allele distance within the range of 0–6. The corresponding SNP differences were 0–12. Additionally, the SNP analysis showed highly congruent results between Illumina and Ion Torrent with differences between 0 and 1.

### Analysis results for CLC

All 19 isolates per the including sites A–I were compared regarding single-nucleotide variant comparisons using CLC and resulted in the same five defined clusters as defined by JASEN and 1928 platform (Figs2^3^). Interestingly, isolates sequenced with the Ion Torrent technology (site A and site I) were closely situated to each other in all the clusters (I–V), differentiating these isolates from the remaining isolates sequenced with the Illumina technology.

**Fig. 5.**
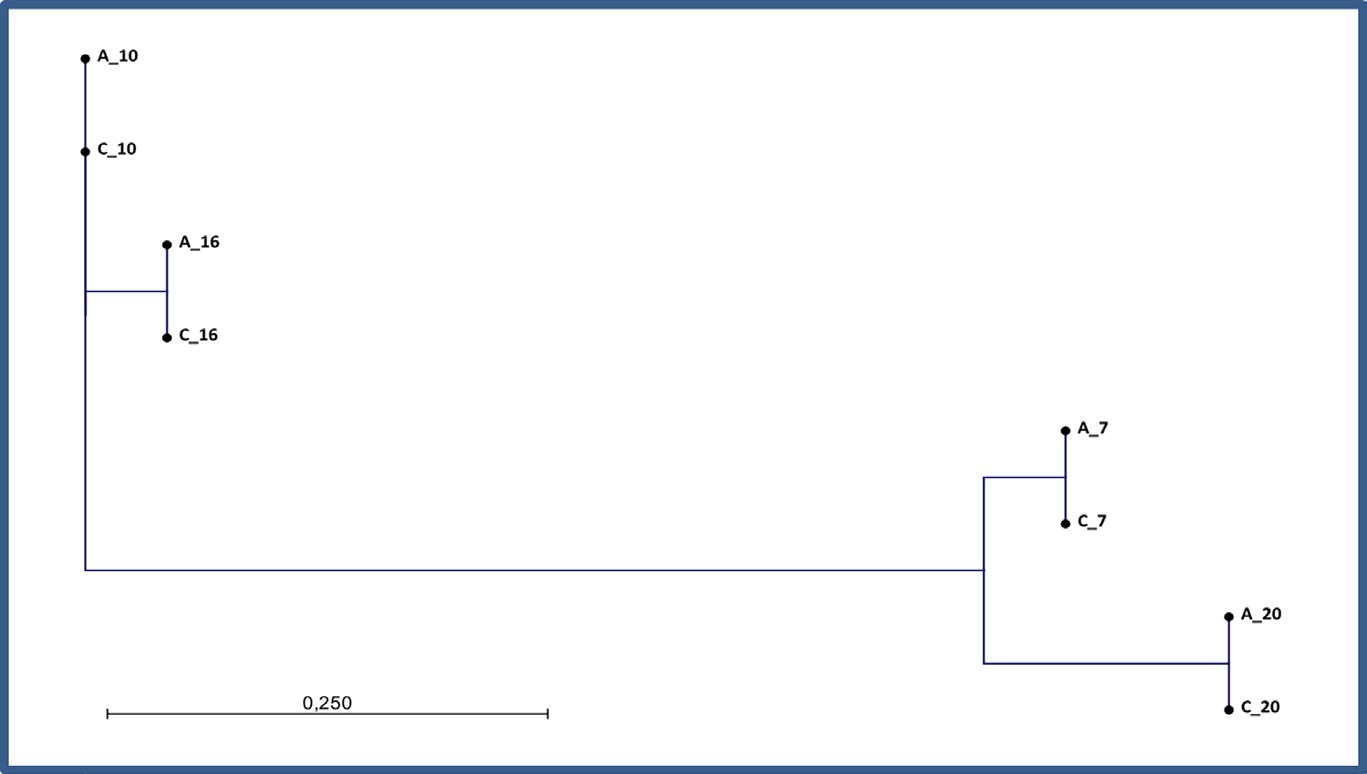
A pairwise SNP analysis was performed (CLC, Qiagen) by mapping the sequences of cluster I (containing the isolates 7, 10, 14 and 20) from site A (Ion Torrent) and site C (Illumina) to the internal reference A_7. The sequenced isolates differ in total 0–15 SNPs within the cluster.

When analysing individual clusters separately, including all the sequences from site A–I, and with the sequence reference *S. aureus* ASM1346v1 (GCF_000013465.1), the sequences generated by the different technologies result in many SNPs between Illumina and Ion Torrent. For cluster I: 0–47 SNPs, cluster II: 0–48 SNPs, cluster III: 0–38 SNPs, cluster IV: 0–95 SNPs and cluster V: 0–97 SNPs (not shown). However, when analysing the individual clusters using an isolate within a specific cluster as a reference, all isolates within cluster I–V were close to one another, regardless of sequence technology, and differed with less than seven SNPs in NJ pairwise comparison (Fig. 5).

## Discussion

In this study, the standardized bioinformatical pipelines enabled multi-centre comparisons with good results, although the participating laboratories used different workflows and sequencing technologies. Whole-genome sequence data from 19 out of 20 MRSA samples produced by nine Swedish hospital laboratories were comparable, while one isolate was excluded due to contamination. The sequences were analysed by three different bioinformatic platforms: JASEN, 1928 and CLC. By using different methods for cgMLST analysis (JASEN and 1928) and SNP analysis (1928 and CLC), the performance of the methods, used separately and in combination, could be evaluated. JASEN and CLC analyses identified the same five clusters in Illumina and Ion Torrent data, respectively. The additional seven isolates were all unrelated and unique to all analysis platforms.

The cgMLST method is robust, which is well-suited to handle large datasets, and provides results with a comprehensive overview of clusters as well as great discriminatory power [26,28]. This can be followed by a more specific and detailed SNP analysis of clusters for further confirmation of strain identities [29]. However, SNP analysis is dependent on reference genomes that are closely related to the set of isolates being compared. Furthermore, the core SNPs shared between isolates decrease with the addition of unrelated samples, making them less sensitive when comparing larger datasets. Coolen *et al*. conducted a multi-centre study, where the same FASTQ data from two datasets of *Klebsiella pneumoniae* and Vancomycin-resistant *Enterococcus faecium*, respectively, were analysed by 13 participants’ internal bioinformatics pipelines. The study revealed discrepancies between centres and their outbreak clustering from the same sequencing data. Even when almost identical bioinformatic workflows were used, the stringency of the filtering impacted the results [17]. This highlights the importance of having a harmonized and standardized pipeline to obtain comparable results.

Performing *de novo* assembly by JASEN, the sequences from the Ion Torrent sequencing technology were not as good as the sequences produced by Illumina regarding the core coverage. Care must be taken when comparing sequences generated by Illumina and Ion Torrent. Ion Torrent-generated sequences are often affected by homopolymer indels that potentially cause an increase in artificial allele differences and make *de novo* assembly more difficult. The number of missing loci in the cgMLST by JASEN (Fig. 1b) directly correlates with the discriminatory power a sequence will have compared to other sequences. This was also shown by a multi-site German study that compared the consistency, accuracy and reproducibility of WGS short-read sequencing between ten laboratories involved in food safety [30]. Here, the cgMLST and SNP analyses were compared using one uniform pipeline, but sequencing covered four different types of Illumina-sequencing platforms (MiSeq, NextSeq, iSeq and NovaSeq) and one Ion Torrent-sequencing instrument (S5). In agreement with our study, homopolymer-related differences between Illumina and Ion Torrent results were obtained when using *de novo* assembly and the ChewBBACA pipeline, whereas the SNP analysis results were not affected. However, as long as the sequence depth is sufficient, the sequencing platform is used to provide adequate discriminatory power. Those differences between the technologies were not seen in 1928 since it is based on k-mer-based cgMLST analysis and hence assembly-free.

This study shows that defining a strict cut-off for a cluster is not a trivial task when different sequencing technologies are used, especially if technology differences are not accounted for in the analysis pipeline. The cgMLST analysis of JASEN showed that the clustering of the Illumina data was consistent, while the Ion Torrent reads with a coverage <30× failed to cluster accordingly due to missing data and reduced discriminatory power. In the CLC SNP analysis (Fig. 3b), the samples clustered together into the five defined clusters of this study but with >20 genetic differences between the sequences of different technologies. However, when using pairwise comparisons and a reference within the cluster, <20 SNPs (excluding recombination events) were clearly seen (Fig. 5) [10]. Also as seen in Fig. 3b, sequences generated by Ion Torrent technology cluster in proximity regardless of site. In addition, libraries constructed with Nextera XT cluster near each other (sites B and D) in the CLC analysis (Fig. 3b). This correlates according to the lower evenness of coverage with Nextera XT. Sequences of site F, with an in-house Nextera library construction method, also reflect a lower sequence quality and cluster with this group.

When using the k-mer-based cgMLST analysis pipeline of 1928 Diagnostics, a cut-off for cluster belongings could here be defined to less than six allele differences, independent of sequencing technology and a varied sequencing depth between the sites, which is also in congruence with what has been seen earlier [20,23]. This cut-off value can be compared with Lagos *et al*., who suggested that an estimated genomic variation rate of 2.0–5.8 cgMLST alleles or SNPs (excluding recombination events) per year could be used as a guideline for clinical laboratories in surveillance and outbreak investigations. However, it should also be considered that the estimated genomic variation rate might vary in different MLST sequence types, as shown by Lagos *et al*., as well as the choice of reference and analysis method used due to the overall variability and suitability they possess [18].

In the future, datasets of well-defined isolates can be used to facilitate the setup and validation of WGS for additional species at different laboratories. Broadly implementing WGS in healthcare across the country will strengthen precision diagnostics and in turn increase the possibility of mapping the spread of infectious diseases and detecting antibiotic resistance. Using pairwise SNP comparisons between isolates as a strategy – in combination with clinical epidemiological data of an outbreak investigation – ensures the maximization of discriminatory power in individual cases. However, this requires a national distribution of well-optimized protocols and joint technical solutions with automated workflows for bioinformatics analysis. In Sweden, this national genomic platform is under development within our national consortia, GMS, and will in the future be the basis for real-time sharing of methods and results between regions affected by outbreaks. Joint national solutions also facilitate international comparisons, as shown to be of great importance by the recent SARS-CoV-2 pandemic.

To get advantage of real-time sharing of data, within a national consortium, it would be beneficial to reduce the sequencing time. A possible next step would be to use the current sample collection to validate sequencing technology with faster turn-around time like Oxford Nanopore Technology (ONT). ONT sequencing has previously been associated with high error rates compared to sequencing technologies like Illumina and Ion Torrent [31], but with the latest revisions in chemistry and base calling, ONT has been reported to give significantly improved quality, making it promising for use in outbreak investigations [32].

In conclusion, a national consortium with joint IT infrastructure and analysis pipelines that handles sequences generated from different sequencing platforms in real time would facilitate outbreak investigations and transmission routes. MRSA isolates, whole-genome sequenced by different laboratories and technologies, and analysed by 1928 Diagnostics, produced comparable results for outbreak clustering for both cgMLST and SNP. In contrast, JASEN and CLC, with the setup used in this study, were best suited to analyse Illumina and Ion Torrent data, respectively. However, the JASEN is in progress to include optimal analysis for more sequencing technologies. In the future, real-time share of data and a joint analysis within the national GMS consortium together with other sequence technologies, such as long-read sequencing techniques with faster turn-around-time, will further facilitate future investigations of outbreaks and transmission routes.

## supplementary material

10.1099/mgen.0.001331 Uncited Supplementary Material 1.

10.1099/mgen.0.001331 Uncited Supplementary Material 2.

## Abbreviations

cgMLST: core genome multilocus sequence typing
IQR: interquartile range
MRSA: methicillin-resistant *Staphylococcus aureus*
NGS: next-generation sequencing
NJ: neighbour-joining
ONT: Oxford Nanopore Technology
QC: quality checked
WGS: whole-genome sequencing
