# Supplementary material for: Evaluation of nationwide analysis surveillance for methicillin-resistant Staphylococcus aureus within Genomic Medicine Sweden
Source: Microb Genom. 2025 Jan 27;11(1):001331. doi: 10.1099/mgen.0.001331 (PMC11893271; doi:10.1099/mgen.0.001331)
Supplement: Uncited Supplementary Material 1. [file mgen-11-01331-s001.pdf]

Supplementary Material

Supplementary Table S1. Characteristics and outbreak definition of the MRSA strains from the national collection were defined by the Public Health Agency of Sweden (PHAS).

**Year**	**ST1 t127**	**ST5 t002**	**ST6 t304**	**ST22 t223**	**ST30 t021**	**ST45 t2187**	**ST59 t437**	**Strains (n)**	**Cluster* yes/no**	***Clusters and unrelated defined in study (isolate)***
2016-2017							2	2	yes	***IV*** *(13, 15)*
2017							1	1	No	*(2)*
2017		2						2	yes	***III*** *(6, 19)*
2017		1						1	no	*(11)*
2017					2			2	yes	***V*** *(4, 8)*
2017	2							2	yes	*** (3)*
2016	1							1	no	*(17)*
2018			2					2	yes	***II*** *(5, 14)*
2017-2018				2				2	no	*(9, 12)*
2018						1		1	no	*(18)*
2018	4							4	yes	***I*** *(7, 10, 16, 20)*
**Total sum**	**7**	**3**	**2**	**2**	**2**	**1**	**3**	**20**		

*Clusters are defined by Public Health Agency of Sweden (PHAS).
**Panton-Valentine leukocidin (PVL) positive strains. All the additional strains are PVL negative.
*** Due to a contaminated isolate this cluster defined by PHAS could not be defined in the study.

Supplementary Table S2. Culture procedures of the 20 MRSA isolates from the Public Health Agency of Sweden (PHAS) at site A-I.

Site	Date of delivery	Culturing media	Culture procedure	Colonies for DNA extraction[c]
A	2019-04-07[a]	Blood agar	Re-culturing of a few single colonies	⅓ blue loop
B	2019-04-07 [a]	Blood agar	No re-culturing	white loop
C	2019-04-02	Blood agar	No re-culturing	blue loop
D	2019-04-02	Blood agar	Re-culturing only if suspicion of contamination	white loop
E	2019-04-02	BD Mueller-Hinton II agar	Re-culture from one colony	⅓ blue loop
F	2019-04-07 [a]	Blood/SAID agar	Re-culturing	white loop
G	2019-11-06[b]	CHROM-agar MRSA plates	No re-culturing	blue loop
H	2019-04-02	Blood agar	Re-culturing	A few separate (pure) colonies
I	-	Blood agar	No re-culturing	white loop

[a] Delivery re-cultured from first culturing episode 2019-04-02 at PHAS
[b] Cultured from freeze tube 2019-04-02
[c] The inoculation loops used were 1µl (white loop) and 10µl (blue loop)

Supplementary Table S3. A summary of methods used for whole-genome sequencing of MRSA at the participating sites.

SITE	DNA EXTRACTION	LIBRARY PREPARATION	SEQUENCING SYSTEM	DATA ANALYSIS
**A**	Enzymatic treatment, Magna Pure Compact (Roche)	Ion Xpress™ Plus Library Kit, (ThermoFisher)	Ion Torrent S5 (ThermoFisher)	CLC Genomics Workbench (Qiagen)
**B**	Enzymatic treatment, EZ1 Advanced XL (Qiagen)	Nextera XT DNA Library Preparation kit (Illumina)	MiSeq v3 (Illumina)	Java script, Spades, Ridom SeqSphere Spades assembly (Ridom GmbH)
**C**	Pre-heating, EZ1 Advanced XL (Qiagen)	QIAseq FX DNA Library Kit (Qiagen)	MiSeq (Illumina)	CLC Genomics Workbench (Qiagen), BioNumerics (Applied Maths)
**D**	Enzymatic treatment, Blood and Tissue (Qiagen)	Nextera XT, half volume (Illumina)	MiniSeq (Illumina)	*In-house* pipeline with QC, cgMLST, MLST, virulence, ChewBBACA and grapetree
**E1**	QiaSymphony (Qiagen)	Illumina DNA prep (Illumina)	MiSeq (Illumina)	Ridom SeqSphere, Velvet assemblage (Ridom GmbH, Germany),1928 Diagnostics
**E2**	QiaSymphony (Qiagen)	Illumina DNA prep (Illumina)	NextSeq 500/550 (Illumina)	Ridom SeqSphere, Velvet assemblage (Ridom GmbH),1928 Diagnostics
**F**	Enzymatic treatment, EZ1 Advanced XL (Qiagen)	Nextera *in-house* (Illumina)	HiSeq or NovaSeq (Illumina)	microsalt and 1928 Diagnostics
**G**	PureLink Microbiome DNA Purification kit (INVITROGEN)	Nextera DNA Flex Library Prep kit (Illumina)	MiSeq (Illumina)	Bcl2fast2 and 1928 Diagnostics
**H**	Magna Pure Compact (Roche)	Nextera XT DNA Library Preparation kit (Illumina)	MiSeq (Illumina)	Geneious and Velvet. Gegenees, ResFinder with MLST (Center for Genomic Epidemiology)
**I**	Enzymatic treatment, PSS, MagLEAD (Precision System Science)	Ion Xpress™ Plus Library Kit, Library builder (ThermoFisher)	Ion Torrent S5 (ThermoFisher)	*In-house* pipeline (BactTyper) based on CLC Assembly Cell; and diverse program

Appendix S1. Whole genome sequencing procedures performed at each site (A-I)

S1-A. Whole genome sequencing of bacterial isolates – site A

Culturing and DNA extraction

The 20 MRSA strains were cultivated on blood agar plates supplemented with horse blood at 37°C over night, and a few standalone fresh (not more than 3 days) colonies suspended in PBS to McFarland 5. Genomic DNA was extracted using the following in-house protocol; 1 ml was concentrated by centrifugation at 7000 g for 5 min and suspended in 320 µl Low-TE buffer (1mM Tris, 0.1mM EDTA, pH 8.0) with Triton X-100 (1.2 %). Staphylococcus sp. enzyme mix [25 µl lysozyme (4*106 U), 15 µl lysostaphin (0.5U μl^{-1}) and 15 µl mutanolysin (1U μl^{-1})] was added at 37 °C for 30–45 min incubation. Then proteinase K [20 µl (20 mg ml^{-1}), 56 °C, 1h] was added, followed by RNaseA [5 µl (100 mg ml^{-1}), room temperature, 5 min]. MagNA Pure Compact (Roche) was used with the MagNA Pure Compact Nucleic Acid Isolation kit I (Roche).

WGS

DNA quantification was done using Nanodrop ND1000 and Qubit 2.0 (both Thermo Fisher Scientific). For library construction Ion Xpress Plus Fragment Library kit for AB Lib Builder system was used with 300 ng gDNA, in AB Library Builder™ System, Thermo Fisher. The libraries were further whole genome sequenced (WGS) in Ion S5™ System, Thermo Fisher.

Analysis

After WGS, trimming, assembly and single-nucleotide polymorphism (SNP) analysis was performed with the CLC Genomics Workbench 12 including the Microbial Genomics Module (Qiagen Bioinformatics). The reference Staphylococcus aureus ASM1346v1 (GCF_000013465.1) was used for mapping the sequence reads followed by local realignment (mapping parameters: match score 1, mismatch cost 2, and a linear gap cost for insertions/deletions 3); variant calling parameters: Ignore broken pairs, ignore specific matches (reads). Coverage and count filters; Minimum coverage=10, Minimum count= 5 (Illumina), Minimum count=10 (Ion Torrent), Minimum frequency=90%). Noise filters =default settings for Illumina. A noise filter was used to take the quality scores of the surrounding bases into account (parameters: neighbourhood radius 5 bp, central quality 20, minimum neighbourhood quality 15). Reads shorter than 20 base pairs were filtered out and a neighbour-joining (NJ) algorithm was used for the SNP analysis (parameters: minimum coverage required in each sample 10, minimum coverage percentage of average required 10, prune distance 20, minimum z-score required 1.96). Isolates differing 20 SNPs or less, are concluded to derive from the same strain. At a difference of 20-40 SNPs, isolates descend possibly from the same strain. Common source of infection cannot be excluded.

S1-B. Whole genome sequencing of bacterial isolates – site B

Culturing and DNA extraction

From the 20 Frozen MRSA isolates a full inoculation-loop was plated on blood agar plates and cultivated on blood agar plates at 37 °C overnight. Without re-culturing several colonies were picked using a white 1µl inoculation loop, filling the loop with material. Colonies were then resuspended in a mixture of 180 µl G2 buffer (Qiagen AB, Hilden, Germany) and 20 µl lysozyme (50 mg/ml) (Sigma-Aldrich, St. Louis, MO) and incubated at 37 °C for 60 min. Nucleic acid was extracted using EZ1 Advanced XL, and the EZ1 tissue DNA kit (Qiagen). The sample input volume was set to 200 µl, and the samples were eluted in 50 µl molecular grade water (GE Healthcare Life Sciences, Utah, USA).

WGS

The DNA concentration was measured using Qubit ® dsDNA HS Assay system (Thermo Fisher Scientific, Waltham, MA). Samples were diluted in two steps to a final concentration of 0.2 ng/µl and 5 µl was used as input material for library preparation**.** Nextera XT DNA Library Preparation kit (Illumina Inc., San Diego, CA) was used to generate libraries according to the Nextera XT DNA Library Prep Kit protocol (#15031942 v01, Illumina Inc.). Library dilution, pooling, normalization and preparation of Phix was performed according to the “Denature and Dilute Libraries Guide” (15039740 v10, Illumina), 1 % Phix was added to the MiSeq run. Libraries were sequenced as 20-plexes using a Miseq (Illumina Inc.), 2*251 bp paired-end sequencing, and Miseq v3 chemistry (Workflow: Generate FASTQ, Application: FASTQ only, Assay: Nextera XT, Chemistry: Amplicon), the data were base called and demultiplexed on the instrument.

Analysis

FastQC Java script was used as a tool for assessing the sequence quality. Cluster density was 732 k/mm2, average Q30 was 82,3 %. Sequences were assembled using Spades in Ridom SeqSphere+ (Ridom GmbH, Münster, Germany). Core genome multilocus sequence typing (cgMLST) was used for type assignment and phylogenetic analysis.

S1-C. Whole genome sequencing of bacterial isolates – site C

Culturing and DNA extraction

Bacterial strains were cultured on blood agar plates overnight (no re-culturing), and 10 µl of cultured bacteria were suspended in 200 µl G2 buffer from the EZ1 DNA Tissue Kit (Qiagen). Samples were vortexed for 10s, incubated at 95°C and 350 rpm for 15 min, and genomic DNA extraction was then performed using the EZ1 DNA Tissue Kit with EZ1 Bacteria settings and an elution volume of 50 µl.

WGS

Samples were quantified using the Qubit dsDNA High Sensitivity Kit (Thermo Fisher Scientific), and 20 ng of DNA was used as starting material for library preparation. Libraries were built using the QIAseq FX DNA Library Kit (Qiagen) according to the manufaturer's instructions, with a fragmentation time of 8 min and a PCR of 8 cycles. After quantification using QIAxcel Advanced High Resolution (Qiagen) and Qubit dsDNA Broad Range Kit (Thermo Fisher Scientific), 10 pM libraries were sequenced on the MiSeq platform (Illumina) with 2 x 300 bp paired-end reads.

Analysis

Data analysis was performed in CLC Genomics Workbench v. 9.5.4 with the Microbial Genomics Module v. 1.6.2 (Qiagen). A custom pipeline was built that included *de novo* assembly with the CLC metagenome assembler, multi locus sequence typing (MLST) by mapping against pubMLST schemes (1), and determination (by K-mer spectra) of, and mapping to, the closest NCBI reference genome. Assembled data was searched for the PVL gene and for antimicrobial resistance genes against the ResFinder database (2), and the spa gene was extracted using virtual primers*. Spa typing was performed in BioNumerics (Applied Maths) against the Ridom SpaServer (http://www.spaserver.ridom.de).Read mappings were used for variant calling with the following thresholds to call a variant: depth of coverage ≥ 20x, variant frequency ≥ 90%, Phred score ≥ 20 in the variant position and Phred score ≥15 in the +/- 5 bp neighborhood. Multinucleotide variants were excluded. For each group of samples with the same spa type, a quality filter was then applied that retained variants with a sequencing depth of ≥ 20x in all samples and a distance ≥ 10 bp to the next variant. The resulting single nucleotide variants were then used to create a tree based on genetic distances (Figure S1-C). Guided by previous studies of *S. aureus* (3), isolates with a distance of ≤ 15 variants were suspected to constitute an outbreak.

* 5'-TAAAGACGATCCTTCGGTGAGC-3' and 5'-CAGCAGTAGTGCCGTTTGCTT-3'

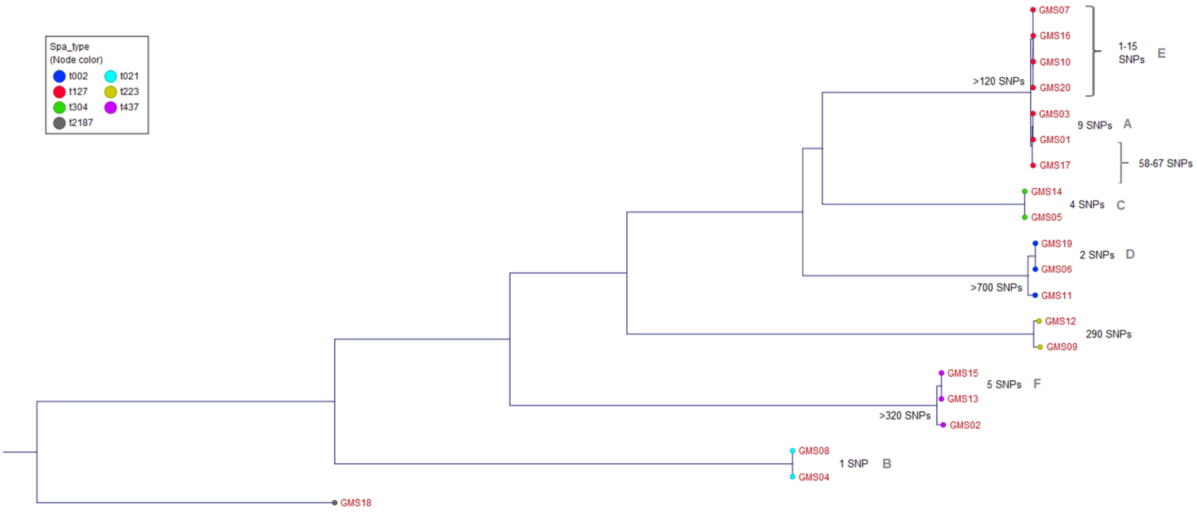

Figure S1-C. A pairwise single nucleotide variant tree analysis constructed in CLC (Qiagen).

S1-D. Whole genome sequencing of bacterial isolates – site D

Culturing and DNA extraction

MRSA isolates were cultured on blood agar plates at 37 °C over-night. Re-culturing was only performed if the colonies appeared to be non-pure. Of each sample, one filled white loop (1 µl) of cultured bacteria was suspended in 98 µl of lysis buffer (20 mM Tris, 2 mM EDTA, 1.2% Triton-X in ultra-pure distilled water) with addition of 72 µl Lysozyme (50 mg/ml) and 10 µl Lysostaphin (100 µg/ml) and incubated at 37°C for 30 min. The extraction was then performed using the DNeasy Blood & Tissue Kit (Qiagen).

WGS

Samples were quantified using the Qubit dsDNA High Sensitivity Kit (Thermo Fisher Scientific), Samples were diluted to 0.2 ng/µl and 5 µl was used as input material to Nextera XT (Illumina), used at half volume reactions. Sequencing was done on MiniSeq 300 cycles (Illumina) run as paired end 2x150.

Analysis

In house pipeline (precursor to JASEN) based on assembly with SPAdes and mapping with BWA MEM. The pipeline consists of a de novo assembly using SPAdes 3.11.1 using "-k 21,33,55,77 --only-assembler" where raw reads are mapped back using bwa mem 0.7.15. The detection and removal of polymorphic sites by finding assembly errors was done by using freebayes. The pipeline includes typing for multi locus sequence typing (MLST) using the seven loci as well as a core genome MLST (cgMLST) using ChewBBACA 2.0.5 and a cleaned-up de novo assembly. The cgMLST scheme according to Leopold et al was used (4). Idenification of virulence and resistance genes was done using Ariba 2.11.1 with the VFDB database. Species/contamination check was done by using Kraken 1 with the prebuilt minikraken database, postprocessing for abundance was done with Bracken. De novo assembly QC was performed using Quast and CP000046.1 as a reference genome. Genome coverage was done by mapping raw reads to CP000046.1 using a bed file to mask out everything that is not a gene in the cgMLST scheme. The visualisation was done with Grapetree.

S1-E. Whole genome sequencing of bacterial isolates – site E

Culturing and DNA extraction

MRSA isolates were subcultured on BD Mueller-Hinton II agar (Becton Dickinson and Company, Sparks, MD, USA), re-cultured from one colony. For DNA extraction 1/3 of a blue loop (10 µl) of cultured bacteria were suspended in 0.85% 1,5 ml NaCl and 800 µl were extracted with Qiasymphony DSP Virus/pathogen MIDI kit including RNAase degradation and exchange of elution buffer to Tris-HCl.

WGS

DNA was quantified using a Qubit fluorometer (Termo Fisher, Waltham, MA). Purity 260/280 and 260/230 was measured using a NanoDrop 1000 (Termofsher) and the fragmentation of the samples was determined using the DNA integrity number (DIN) by using a TapeStation 4200 system (Agilent, Santa Clara, CA). Libraries were built using the Nextera DNA prep LibraryPrep kit (Illumina Inc., San Diego, CA) according to the manufaturer's instructions. Sequencing was performed using an Illumina MiSeq™ (Illumina) with MiSeq™ Reagent Kit v3 (600 cycles) or an Illumina NextSeq ™ (Illumina) with NextSeq 500/550 Mid Output Kit v2.5, 300 Cycles, according to the manufacturer's instructions.

Analysis

The genome sequencing data was processed by the sofware Ridom SeqSphere+5.0.0 (Ridom GmbH, Münster, Germany) and the cloud-based 1928 platform (1928 Diagnostics, Gothenburg, Sweden) (5). For Ridom SeqSphere+ (6) the reads were de novo assembled using Velvet integrated in Ridom SeqSphere+32 using default settings: reads were trimmed until the average base quality of 30 was reached in a window of 20 bases. The samples were aligned to the COL reference sequence analysed by cgMLST using the sofware Ridom SeqSphere+scheme based on 1,861 genes (7, 8). More than 50-fold average coverage with an average read length of>200 bp, and percentage good targets for cgMLST≥97% was considered acceptable sequence quality for further data analysis. A phylogenetic tree was constructed in SeqSphere+ using their neighbour-joining tree algorithm (Fig. 1) as well as a Minimum Spanning Tree (MST, Fig.2). Additional information was extracted regarding the ST, CC, spa and CT (Complex Type in cgMLST). The 1928 platform's SNP analysis pipeline and the 1928 platform's cgMLST method uses a custom developed allele calling algorithm based on an alignment free k-mer approach.

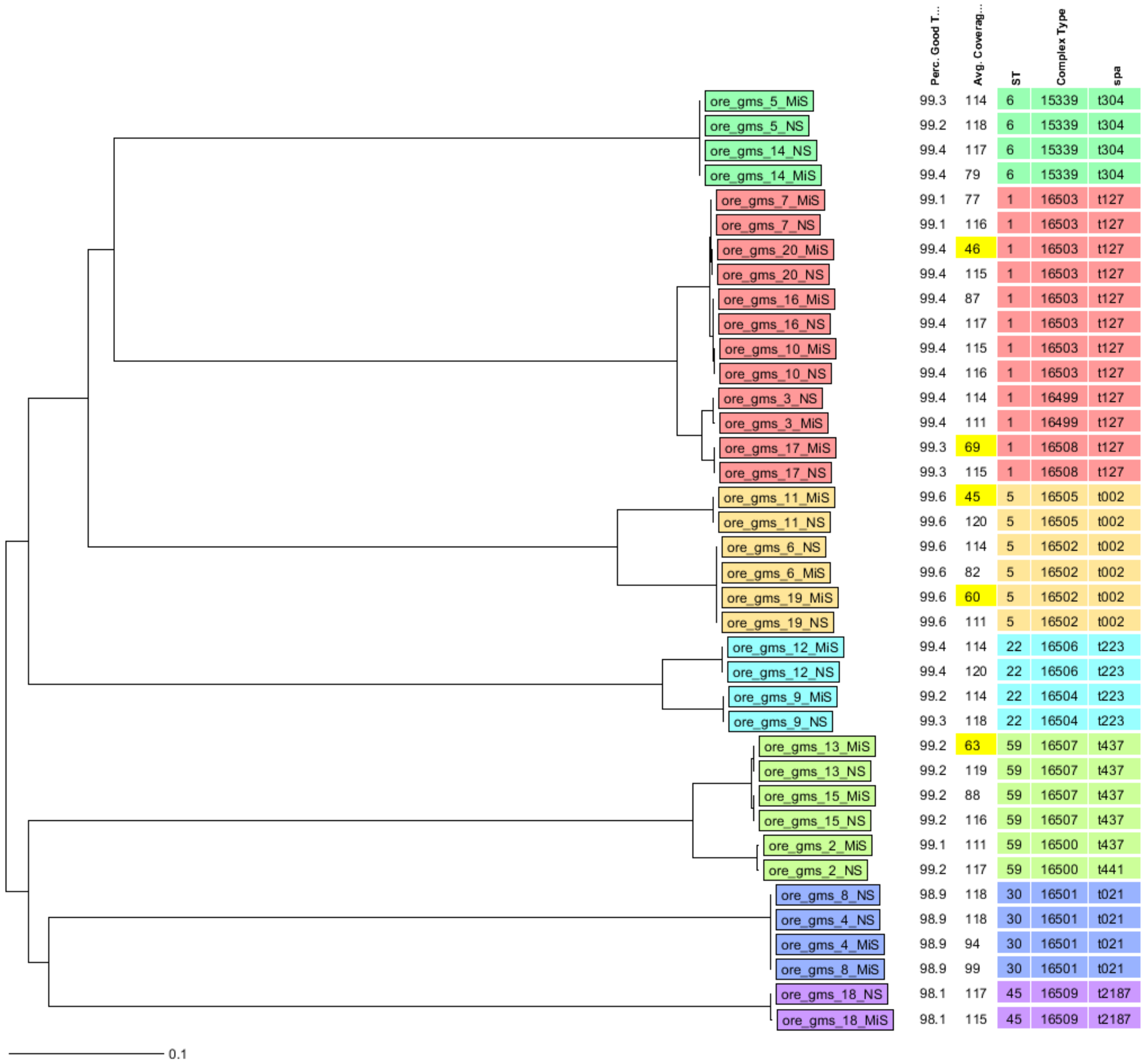

Figure S1-E. Phylogenetic tree, constructed in Ridom SeqSphere+, using neighbour-joining tree algorithm.

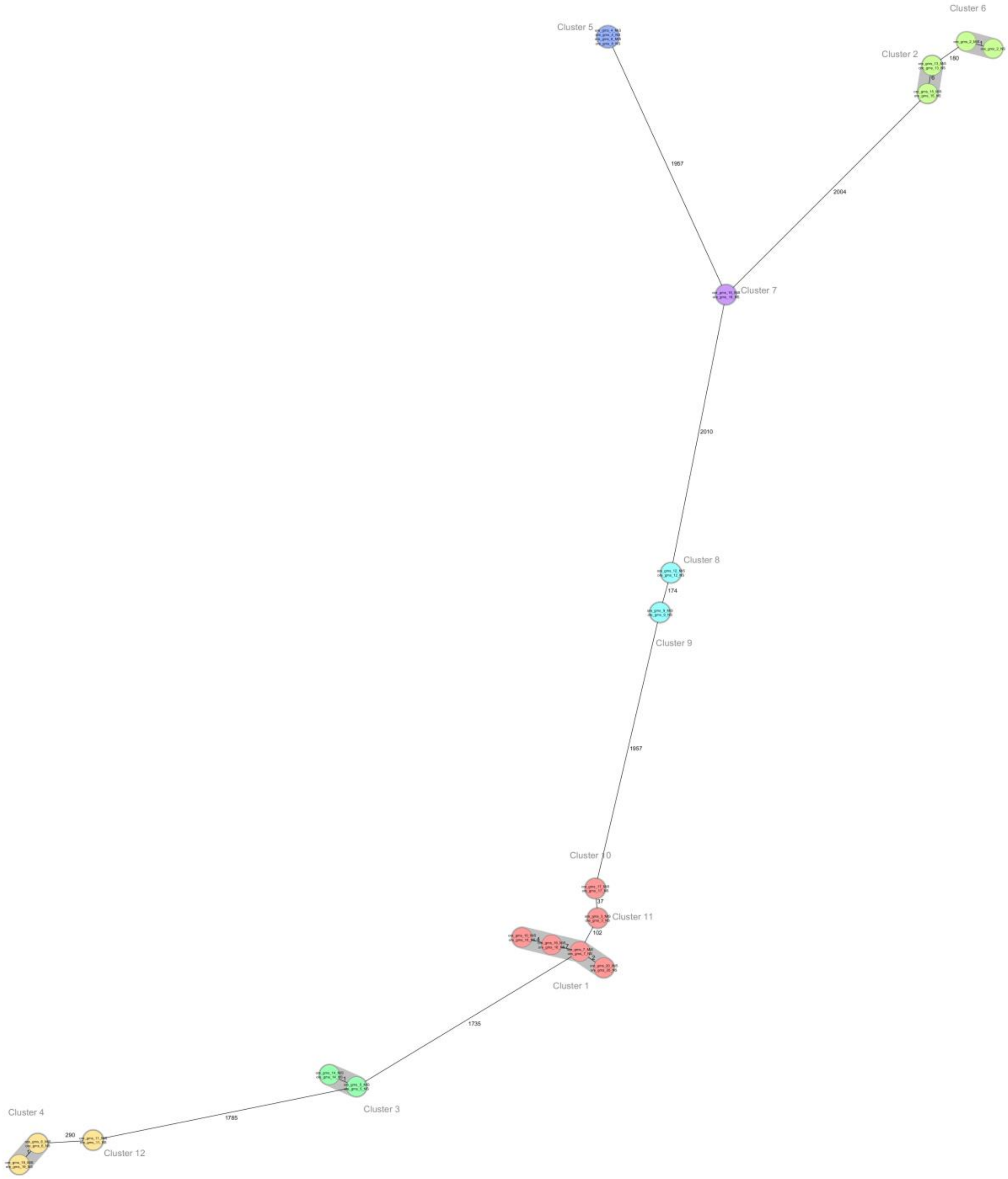

Figure S2-E. Minimus Spanning Tree (MST), constructed in Ridom SeqSphere+.

S1-F. Whole genome sequencing of bacterial isolates – site F

Culturing and DNA extraction

A loop of each MRSA isolates was cultured on Blood or SAID agar, followed by re-culturing of 1 µL of colonies (white loop). Colonies (1 µL) were then re-suspended in 180 µL Buffer G2 and enymatic pre-treated with Lysozyme (>6 000 units) and Lysostaphin (>2 000 units), followed by DNA extraction using EZ1&2® DNA Investigator® Kit on a EZ1 Advanced XL (Qiagen).

WGS

Genomic DNA was quantified using Quant-iT dsDNA High-Sensitivity assay kit (ThermoFisher) and normalised to 2 ng/ul. One ul of normalised genomic DNA was used in the tagmentation reaction using Illumina Tagment DNA TDE1 Enzyme and Buffer Kit (Illumina) to yield fragments of approximately 150 bp. Amplification of tagmented library was carried out using 11 cycles of PCR with dual indexed primers (Integrated DNA Technologies). PCR products were purified using Seramag beads (9). The obtained libraries were quantified using Quant-iT dsDNA High-Sensitivity assay kit (ThermoFisher) and selected samples were run on TapeStation D1000 HS assay (Agilent). The libraries were pair-end (2x150 bp) sequenced on a HiSeq2500 or NovaSeq 6000 to average 7 M read pairs (range 3-14, SD=2).

Analysis

Base calling and demultiplexing was done using Casava, without allowing for any mismatches in the index sequence and analysed using microsalt version 3.3.4. Analysis for cgMLST and resistance was performed with 1928D.

Samples

Ticket #609108 and sample ACC5614A3

Table S1-F. Quality control criteria

Parameter	FAIL criteria	WARNING criteria	Comment
Total Reads	<= 70% of apptag order	<=75% of apptag order	see below *
Total Reads (Negative Control)	>=20% of apptag order	>=10% of apptag order	Only for negative controls by Clinical Genomics
Mapped Rate	<=30%	<=50%	
Duplication rate	>=80%	>=20%	
Median Insert Size	<=100 bp	<=140 bp	
Average Coverage	<=10x	<=100x	
%BP > 10x Coverage	<=75%	<=85%	
%BP > 30x Coverage	-	<=70%	
%BP > 50x Coverage	-	<=50%	
%BP > 100x Coverage	-	<=20%	

S1-G. Whole genome sequencing of bacterial isolates – site G

Culturing and DNA extraction

Bacterial strains were cultured on CHROMagar MRSA plates for 24h without re-culturing. DNA was extracted using the PureLink Microbiome DNA Purification kit (INVITROGEN) and the manufacturer´s protocol for purification of high-quality microbial DNA from microbial culture and transport media samples (MAN0014332), with the following modifications: 10 colonies of each bacterial strain were resuspended in 500 µl nuclease-free water and then pelleted by centrifugation. Pellets were resuspended in 800 µl S1 Lysis Buffer, mixed with 100 µl S2 Lysis-Enhancer and incubated at 65°C for 10 minutes. Bead beating was performed using the TissueLyser LT instrument (QIAGEN) with two runs for 5 min at 50 1/s oscillation for each sample.

WGS

DNA concentration and 260/280 and 260/230 ratios were assessed using a NanoDrop instrument (ThermoFisher). Sequencing libraries were built with the Nextera DNA Flex Library Prep Kit and Nextera DNA CD Indexes (Illumina) according to the manufacturer's instructions using 300 ng of purified DNA as starting material. Fragment size assessment and quantification of the library were performed using a 2200 TapeStation (Agilent) and Qubit (ThermoFisher) before sequencing on the MiSeq platform (Illumina) with MiSeq™ Reagent Kit v3 (600 cycles).

Analysis

Sequencing data was demultiplexed using bcl2fast2 v2.20 (Illumina) and analyzed with 1928Diagnostics automated pipeline (www.1928diagnostics.com).

S1-H. Whole genome sequencing of bacterial isolates – site H

Culturing and DNA extraction

The bacteria were incubated overnight on blood agar plates (re-culturing only if contamination was visible). Separate colonies were chosen for DNA extraction with the MagNa Pure compact Nucleic Acid Isolation Kit I (Roche, Mannheim, Germany) according to the manufacturer's protocol version 12. The elution volume was 100 µl. DNA quantification was done with Qubit dsDNA High sensitivity kit (Invitrogen).

WGS

The WGS libraries were prepared using Nextera XT DNA Library Preparation kit (Illumina)DNA CD Indexes according to the manufacturer's instructions. The sequencing was performed on a MiSeq platform (Illumina) with MiSeq Reagent Kit v3 2x150 bp paired-end-reads (Illumina.

Analysis

Raw data (. fastq) was imported in Geneious version 8.1.5 (10) and all reads without information, as well reads shorter than 100 bp, were deleted. *De novo* assembly of the genomes was performed by the Velvet plugin (6) running in Geneious. The average nucleotide identity (ANI) was calculated using the Gegenees software version 2.2.1 with a threshold of 20% (11) (Figure S1-H). The corresponding Nexus file was exported and used for constructing the phylogenetic tree in Geneious (Figure S2-H). Resistance genes were determined by ResFinder and Sequence types with the MLST 2.0 service, both provided by the Center for Genomic Epidemiology, Denmark(12, 13).

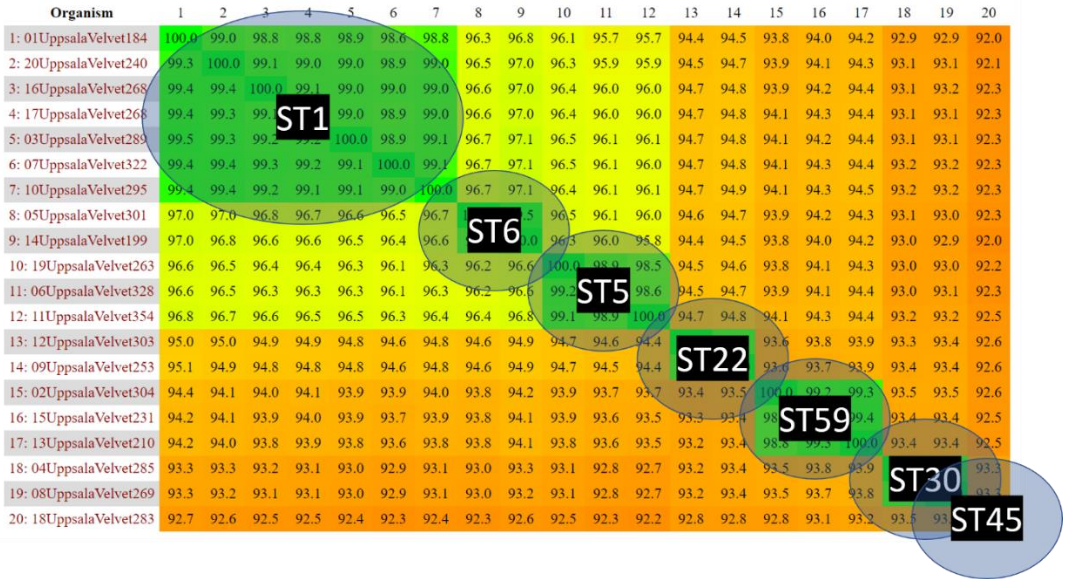

Organism	1	2	3	4	5	6	7	8	9	10	11	12	13	14	15	16	17	18	19	20
1: 01UppsalaVelvet184	100.0	99.0	98.8	98.8	98.9	98.6	98.8	96.3	96.8	96.1	95.7	95.7	94.4	94.5	93.8	94.0	94.2	92.9	92.9	92.0
2: 20UppsalaVelvet240	99.3	100.0	99.1	99.0	99.0	98.9	99.0	96.5	97.0	96.3	95.9	95.9	94.5	94.7	93.9	94.1	94.3	93.1	93.1	92.1
3: 16UppsalaVelvet268	99.4	99.4	100.0	99.1	99.0	99.0	99.0	96.6	97.0	96.4	96.0	96.0	94.7	94.8	93.9	94.2	94.4	93.1	93.2	92.3
4: 17UppsalaVelvet268	99.4	99.3	[illegible]	[illegible]	99.0	98.9	99.0	96.6	97.0	96.4	96.0	96.0	94.7	94.8	94.1	94.3	94.4	93.1	93.1	92.3
5: 03UppsalaVelvet289	99.5	99.3	99.2	[illegible]	100.0	98.9	99.1	96.7	97.1	96.5	96.1	96.1	94.7	94.8	94.1	94.2	94.4	93.1	93.1	92.3
6: 07UppsalaVelvet322	99.4	99.4	99.3	99.2	99.1	100.0	99.1	96.7	97.1	96.5	96.1	96.0	94.7	94.8	94.1	94.3	94.4	93.2	93.2	92.3
7: 10UppsalaVelvet295	99.4	99.4	99.2	99.1	99.1	99.0	100.0	96.7	97.1	96.4	96.1	96.1	94.7	94.9	94.1	94.3	94.5	93.2	93.2	92.3
8: 05UppsalaVelvet301	97.0	97.0	96.8	96.7	96.6	96.5	96.7	[illegible]	[illegible]	96.5	96.1	96.0	94.6	94.7	93.9	94.2	94.3	93.1	93.0	92.3
9: 14UppsalaVelvet199	97.0	96.8	96.6	96.6	96.5	96.4	96.6	[illegible]	[illegible]	96.3	96.0	95.8	94.4	94.5	93.8	94.0	94.2	93.0	92.9	92.0
10: 19UppsalaVelvet263	96.6	96.5	96.4	96.4	96.3	96.1	96.3	96.2	96.6	100.0	98.9	98.5	94.5	94.6	93.8	94.1	94.3	93.0	93.0	92.2
11: 06UppsalaVelvet328	96.6	96.5	96.3	96.3	96.3	96.1	96.3	96.2	96.6	99.2	[illegible]	98.6	94.5	94.7	93.9	94.1	94.4	93.0	93.1	92.3
12: 11UppsalaVelvet354	96.8	96.7	96.6	96.5	96.5	96.3	96.4	96.4	96.8	99.1	98.9	100.0	94.7	94.8	94.1	94.3	94.4	93.2	93.2	92.5
13: 12UppsalaVelvet303	95.0	95.0	94.9	94.9	94.8	94.6	94.8	94.6	94.9	94.7	94.6	94.4	[illegible]	[illegible]	93.6	93.8	93.9	93.3	93.4	92.6
14: 09UppsalaVelvet253	95.1	94.9	94.8	94.8	94.8	94.6	94.8	94.6	94.9	94.7	94.5	94.4	[illegible]	[illegible]	93.6	93.7	93.9	93.4	93.4	92.6
15: 02UppsalaVelvet304	94.4	94.1	94.0	94.1	93.9	93.9	94.0	93.8	94.2	93.9	93.7	93.7	93.4	93.5	100.0	99.2	99.3	93.5	93.5	92.6
16: 15UppsalaVelvet231	94.2	94.1	93.9	94.0	93.9	93.7	93.9	93.8	94.1	93.9	93.6	93.5	93.3	93.4	[illegible]	[illegible]	99.4	93.4	93.4	92.5
17: 13UppsalaVelvet210	94.2	94.0	93.8	93.9	93.8	93.6	93.8	93.8	94.1	93.8	93.6	93.5	93.2	93.4	98.8	99.3	100.0	93.4	93.4	92.5
18: 04UppsalaVelvet285	93.3	93.3	93.2	93.1	93.0	92.9	93.1	93.0	93.3	93.1	92.8	92.7	93.2	93.4	93.5	93.8	93.9	[illegible]	[illegible]	93.3
19: 08UppsalaVelvet269	93.3	93.2	93.1	93.1	93.0	92.9	93.1	93.0	93.2	93.1	92.8	92.7	93.2	93.4	93.5	93.7	93.8	[illegible]	[illegible]	93.3
20: 18UppsalaVelvet283	92.7	92.6	92.5	92.5	92.4	92.3	92.4	92.3	92.6	92.5	92.3	92.2	92.8	92.8	92.8	93.1	93.2	93.5	[illegible]	[illegible]

Figure S1-H. Organism and sequence type determination.

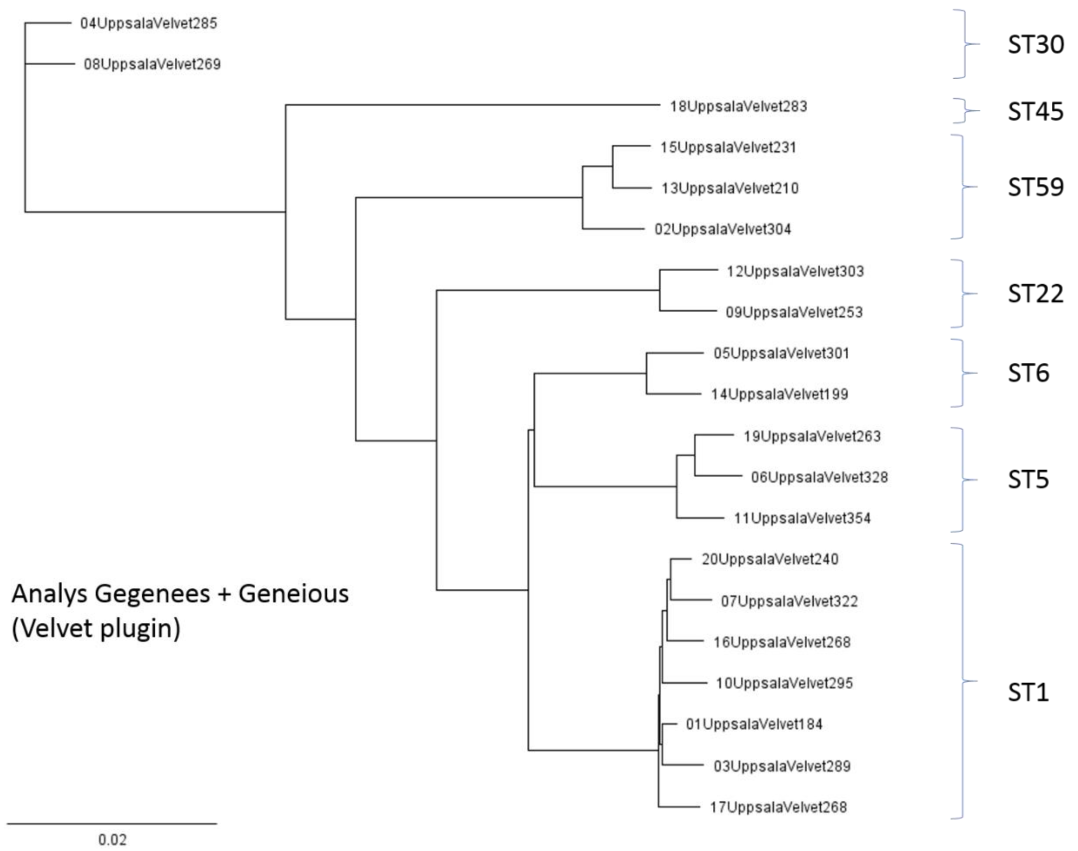

Figure S2-H. Phylogenetic tree.

S1-I. Whole genome sequencing of bacterial isolates – site I

Culturing and DNA extraction
About 10 µl of cultured bacteria were suspended in 180 µl TE-buffer 3 containing 0,1 % Triton, 20 mg/mL Lysozyme with the addition of 1 µl Lysostafin specific for *Staphylococcus aureus*. Samples were incubated 1h at 37˚C (vortexed after 30 minutes). 20 µL Proteinas K were then added to each sample followed by incubation for 1h at 58˚C for (vortexed after 30 minutes). 2 µL 100 mg/mL RNAse A were then added to the samples. After pulse centrifugation DNA was extracted using MagDEA Dx SV REF E1300 on the extraction robot PSS MagLEAD (Precision System Science), elution volume 100 µl. Samples were quantified using Qubit 2.0 Fluorometer with dsDNA High Sensitivity Assay Kit (Thermo Fisher Scientific) with the lower limit of 10ng/ul DNA in a sample to proceed to sequencing.

WGS
The Ion Xpress™ Plus Library Kit (Thermo Fisher Scientific) were used to generate barcoded fragment Ion DNA libraries of 200 ng gDNA per sample. The libraries were pooled and purified by AMPure XP (Beckmann Coulter). After quantification by an in-house RT-PCR assay, 25 ul of 35 pM libraries were used for template preparation and 530 chip loading on an Ion Chef system. The templates were sequenced on an Ion GeneStudio S5 Prime system (Thermo Fisher Scientific).

Analysis
The resulting sequences were analyzed using the in-house pipeline BactTyper; in short, sequence reads were assembled using CLC assembly cell. Following this, the assembly was checked for similarity with an in-house reference database using BLASTn. Genetic markers (species, virulence and antibiotic resistance) were analyzed using in-house implementations of databases by use of BLASTn (e.g. ResFinder, MLST etc.). Within sequence type, whole-genome SNP analysis was performed following the MLST analysis. Additionally, the detected genetic markers were extracted, and raw sequence reads were remapped against them, presenting only markers with coverage above 20x or 10x (MLST). Using the resulting genetic distance from the SNP analysis, a minimum spanning tree was constructed relating the samples to the in-house collection of samples within the ST.

Supplementary References

1. Jolley KA, Bray JE, Maiden MCJ. Open-access bacterial population genomics: BIGSdb software, the PubMLST.org website and their applications. Wellcome Open Res. 2018;3:124.
2. Zankari E, Hasman H, Cosentino S, Vestergaard M, Rasmussen S, Lund O, et al. Identification of acquired antimicrobial resistance genes. J Antimicrob Chemother. 2012;67(11):2640-4.
3. Schurch AC, Arredondo-Alonso S, Willems RJL, Goering RV. Whole genome sequencing options for bacterial strain typing and epidemiologic analysis based on single nucleotide polymorphism versus gene-by-gene-based approaches. Clin Microbiol Infect. 2018;24(4):350-4.
4. Leopold SR, Goering RV, Witten A, Harmsen D, Mellmann A. Bacterial whole-genome sequencing revisited: portable, scalable, and standardized analysis for typing and detection of virulence and antibiotic resistance genes. J Clin Microbiol. 2014;52(7):2365-70.
5. Lagos AC, Sundqvist M, Dyrkell F, Stegger M, Soderquist B, Molling P. Evaluation of within-host evolution of methicillin-resistant Staphylococcus aureus (MRSA) by comparing cgMLST and SNP analysis approaches. Sci Rep. 2022;12(1):10541.
6. Zerbino DR, Birney E. Velvet: algorithms for de novo short read assembly using de Bruijn graphs. Genome Res. 2008;18(5):821-9.
7. Junemann S, Sedlazeck FJ, Prior K, Albersmeier A, John U, Kalinowski J, et al. Updating benchtop sequencing performance comparison. Nat Biotechnol. 2013;31(4):294-6.
8. Weterings V, Bosch T, Witteveen S, Landman F, Schouls L, Kluytmans J. Next-Generation Sequence Analysis Reveals Transfer of Methicillin Resistance to a Methicillin-Susceptible Staphylococcus aureus Strain That Subsequently Caused a Methicillin-Resistant Staphylococcus aureus Outbreak: a Descriptive Study. J Clin Microbiol. 2017;55(9):2808-16.
9. Rohland N, Reich D. Cost-effective, high-throughput DNA sequencing libraries for multiplexed target capture. Genome Res. 2012;22(5):939-46.
10. Kearse M, Moir R, Wilson A, Stones-Havas S, Cheung M, Sturrock S, et al. Geneious Basic: an integrated and extendable desktop software platform for the organization and analysis of sequence data. Bioinformatics. 2012;28(12):1647-9.
11. Agren J, Sundstrom A, Hafstrom T, Segerman B. Gegenees: fragmented alignment of multiple genomes for determining phylogenomic distances and genetic signatures unique for specified target groups. PLoS One. 2012;7(6):e39107.
12. Larsen MV, Cosentino S, Rasmussen S, Friis C, Hasman H, Marvig RL, et al. Multilocus sequence typing of total-genome-sequenced bacteria. J Clin Microbiol. 2012;50(4):1355-61.
13. Bortolaia V, Kaas RS, Ruppe E, Roberts MC, Schwarz S, Cattoir V, et al. ResFinder 4.0 for predictions of phenotypes from genotypes. J Antimicrob Chemother. 2020;75(12):3491-500.
